# Amorphous Fe_2_O_3_ Anchored on N-Doped Graphene with Internal Micro-Channels as an Active and Durable Anode for Sodium-Ion Batteries

**DOI:** 10.3390/nano14110937

**Published:** 2024-05-27

**Authors:** Lin Li, Hui Li, Linxin Liu, Xunchang Yan, Yunze Long, Wenpeng Han

**Affiliations:** 1 Collaborative Innovation Center for Nanomaterials & Devices, College of Physics, Qingdao University, Qingdao 266071, China; 2021023403@qdu.edu.cn (L.L.); liulinxin@bestry-tech.com (L.L.); 2021020305@qdu.edu.cn (X.Y.); 2 State Key Laboratory of Bio-Fibers and Eco-Textiles, Qingdao University, Qingdao 266071, China; lihui28@qdu.edu.cn

**Keywords:** graphene-based macroscopic material, functional composites, energy storage

## Abstract

The reduced graphene oxide (rGO) exhibits outstanding electrical conductivity and a high specific surface area, making it a promising material for various applications. Fe_2_O_3_ is highly desirable due to its significant theoretical capacity and cost-effectiveness, high abundance, and environmental friendliness. However, the performance of these r-GO/Fe_2_O_3_ composite electrodes still needs to be further improved, especially in terms of cycle stability. The composite of Fe_2_O_3_ anchored on N-doped graphene with inside micro-channels (Fe_2_O_3_@N-GIMC) was used to be efficiently prepared. Because the inside channels can furnish extra transmission pathways and absorption websites and the interconnected structure can efficaciously forestall pulverization and aggregation of electrode materials. In addition, N doping is also beneficial to improve its electrochemical performance. Thus, it demonstrates exceptional sodium storage characteristics, including notable electrochemical activity, impressive initial Coulombic efficiency, and favorable rate performance. The optimized Fe_2_O_3_@N-GIMC indicates outstanding discharge capacity (573.5 mAh g^−1^ at 1 A g^−1^), significant rate performance (333.6 mAh g^−1^ at 8 A g^−1^), and stable long-term cycle durability (308.9 mAh g^−1^ after 1000 cycles at 1 A g^−1^, 200.8 mAh g^−1^ after 4000 cycles at 1 A g^−1^) as a sodium-ion battery anode. This presents a new approach for preparing graphene-based high-functional composites and lays a stable basis for further expanding its application field.

## 1. Introduction

As lithium-ion battery (LIB) performance advances, its extensive use in handheld electronic devices, electric cars, and other fields has a profound impact on the development of these fields, thus leading the change in living habits and social development. However, due to the low lithium content in the crust and the uneven distribution, the cost of LIBs has increased rapidly in recent years [1,2]. Therefore, the development of new cheap secondary batteries based on the abundant elements of the earth has become the focus of recent scientific research and industry attention. Among them, sodium-ion batteries (SIBs) based on widely available and low-cost sodium have been widely studied because of their electrochemical working principles similar to LIBs [3,4,5,6,7]. However, due to reasons such as the sluggish electrochemical reaction kinetics and unfavorable structure disintegration caused by the greater ionic radius and higher atomic mass, it is often challenging to secure high specific capacity and superior cycle reliability, thus affecting practical applications [8,9,10,11]. Therefore, it is imperative to identify appropriate anode materials for SIBs with excellent performance through materials’ structural tailoring or exploration of new electrode design principles.

Graphite is a reliable and commonly used commercial anode material for LIBs, with good cyclability and safety [12,13,14]. However, because the radius of Na^+^ is larger than the interlayer spacing (d-spacing) and for other reasons, it is not an ideal anode material for SIBs [15]. Due to the excellent properties such as good conductivity, high specific surface area, and wide electrochemical potential window, reduced graphene oxide (r-GO) has been extensively studied in the field of electrode materials for rechargeable batteries [15,16,17]. In addition to participating in electrochemical reactions as an active material, its excellent electrical conductivity helps to improve kinetics, and good mechanical properties can support the structural integrity of the electrodes during charge discharge cycles. Recent reports indicate that the electrochemical performance of rechargeable batteries based on r-GO strongly depends on its morphology and pore size distribution [3,15,18,19,20]. In our earlier works [21,22], an r-GO film featuring a network of interconnected internal micro-channels was effectively fabricated by integrating electrospinning and electrospray techniques. The findings demonstrate that the LIB utilizing an r-GO film as the anode material exhibits outstanding performance, characterized by a high specific capacity and exceptional cycle stability. This would be attributed to the presence of inner channels, which provide improved transmission pathways and absorption websites, in addition to the interconnected structure that efficaciously mitigates pulverization and aggregation of electrode substances. However, the sodium storage performance of the r-GO film is unsatisfactory, especially due to the low specific capacity [21].

In addition, heteroatom-doping and metal oxide/sulfide composites are also effective ways to enhance the sodium storage capabilities of r-GO-based materials. Because it can improve the electrical conductivity and promote Na^+^ adsorption and insertion, various heteroatoms have been doped into r-GO layers to regulate properties, such as N, P, B, S, and F [3,23,24,25,26,27,28]. Meanwhile, numerous metal oxides/sulfides have been extensively researched as promising electrode materials in SIBs due to their high theoretical specific capacity [29,30,31,32]. Among them, Fe_2_O_3_ is of significant interests because of its high theoretical capacity, low cost, high abundance, and environmental friendliness [33,34,35,36,37,38,39,40,41]. In these studies, some interesting work is to compound Fe_2_O_3_ with r-GO to improve the specific capacity of the material and solve the problem of rapid capacity decay due to the poor electrical conductivity of Fe_2_O_3_ [33,35,36,37]. However, the performance of these r-GO/Fe_2_O_3_ composite electrodes still needs to be further improved, especially in terms of cycle stability.

In this investigation, the combination film of nylon nanofibers, GO flakes, and Fe_2_O_3_ was synthesized using a combination of electrospinning and electrospray techniques. Subsequently, the reduction of GO sheets and the breakdown of nylon nanofibers took place through the process of thermal reduction. The successful formation of the Fe_2_O_3_@N-GIMC composite, where Fe_2_O_3_ is anchored on N-doped graphene with internal micro-channels (Fe_2_O_3_@N-GIMC), was achieved in this investigation. The outstanding performance of the material can be attributed to the increased availability of transmission pathways and absorption sites facilitated by the internal channels, as well as the interconnected architecture, which effectively mitigates the pulverization and aggregation of electrode materials. In addition, N doping is also beneficial to improve its electrochemical performance. It exhibits superior sodium storage properties such as high electrochemical activity, high initial Coulombic efficiency, and good rate performance. The optimized Fe_2_O_3_@N-GIMC indicates staggering discharge capacity (573.5 mAh g^−1^ at 1 A g^−1^), significant rate of achievement (333.6 mAh g^−1^ at 8 A g^−1^), and resilient long-term cycle robustness (308.9 mAh g^−1^ after 1000 cycles at 1 A g^−1^, 200.8 mAh g^−1^ after 4000 cycles at 1 A g^−1^) as a sodium-ion batteries anode. This presents a novel approach to fabricating high-performance composites using graphene and establishes a firm basis for broadening its range of applications.

## 2. Materials and Methods

### 2.1. Materials

A commercially available dispersion of monolayer graphene oxide (GO) in N, N-Dimethylformamide (DMF) solvent was obtained from Gaoxi Technology (Hangzhou, China), with a concentration of 10 mg g^−1^. Detailed technical indicators are shown in Table 1. Before use, the dispersion was reduced to one-third of its initial concentration. Then iron acetylacetonate was added into the prepared graphene dispersion. Formic acid (88%) and iron acetylacetone were purchased from China National Pharmaceutical Corporation (Beijing, China) without further purification. Nylon PA66 (262.35 g mol^−1^) was purchased from Sigma-Aldrich (St. Louis, MO, USA). Then formic acid was used as the solvent to prepare a 16% concentration of nylon solution.

### 2.2. Preparation of the Combined Film of GO Flakes Containing Iron Acetylacetonate and Nylon Nanofibers

Nylon nanofiber film was prepared on aluminum foil by a traditional electrospinning process, which was used as the base material for subsequent experiments. Then, the prepared foundation was securely attached to the roller’s surface, and the nylon solution and the graphene dispersion doped with iron acetylacetonate loaded into different syringes. The infusion rate of the syringes was regulated by individual injection pumps. In contrast, the needle tips of the syringes were linked to a high-voltage power supply capable of producing a DC voltage. The spacing between the needle tip and the drum surface was adjusted to 5 cm and 10 cm, respectively. GO sheets doped with iron acetylacetonate were deposited on the substrate at a feeding rate of 25 μL·min^−1^ and a voltage of 14 kV. At the same time, nylon nanofibers were uniformly dispersed onto the substrate using the electrospinning technique at a rate of 25 μL·min^−1^ and a voltage of 10 kV, while the roller rotated at a speed of 200 rpm. By combining electrospinning and electrospray technology, GO sheets doped with iron acetylacetonate and nylon fiber combined films were prepared successfully. In addition, there is a nylon fiber film on the substrate, which is conducive to the combined film removed from the aluminum foil.

### 2.3. Preparation of Fe_2_O_3_@N-GIMC and Fe_2_O_3_@G

The combined film was removed from the aluminum foil and placed in a tubular furnace. It was thermally annealed under an argon-hydrogen gas mixture to reduce the GO sheet and eliminate the nylon nanofibers, while the iron acetylacetonate was thermally decomposed. During the thermal reduction process, the combined film was slowly heated from room temperature to 500 °C at a pace of 2 °C per minute and maintained at this temperature for 240 min. It was allowed to cool to room temperature and the film removed. After annealing, there was no nylon fiber in the combined film, so the composite of Fe_2_O_3_ anchored on N-doped graphene with internal microchannels (Fe_2_O_3_@N-GIMC) was prepared. If no nylon fiber was added in the preparation process, the composite of Fe_2_O_3_ anchored on graphene with no internal micro-channels (Fe_2_O_3_@G) could be prepared by repeating the spinning and thermal annealing procedures above. According to the mass ratio of iron acetylacetonate (m) and GO dispersion (n) in the precursor solution, the prepared composite material was labeled as Fe_2_O_3_@N-GIMC_m:n and the composite material prepared by not incorporating nylon fiber is labeled as Fe_2_O_3_@G_m:n.

### 2.4. Characterization

The morphologies of the graphene oxide (GO), reduced graphene oxide (rGO), Fe_2_O_3_@G, and Fe_2_O_3_@N-GIMC were analyzed employing a field emission scanning electron microscope (SEM) (FE-SEM, JEOL JSM-7800F, JEOL, Tokyo, Japan). Their compositions were determined via X-ray photoelectron spectroscopy (XPS) (Thermo Escalab 250, Thermo Fisher Scientific, Waltham, MA, USA). The structure was characterized by X-ray diffraction (XRD) (Japan, Smart Lab 3KW, Rigaku, Tokyo, Japan). The morphology was characterized by a Transmission Electron Microscope (TEM) (Japan Corporation, JEM 2100F, JEOL). The types and contents of micro-component elements were characterized by an Energy Dispersive Spectrometer (EDS) (Thermo Fishe, ESCALAB Xi+, Thermo Fisher Scientific).

### 2.5. Electrochemical Experiments

The electrochemical tests of the Fe_2_O_3_@N-GIMC and Fe_2_O_3_@G anodes were carried out in 2032coin cells. The working electrode composition was formulated by mixing active ingredients (80 wt%), Super P (10 wt%), sodium carboxymethylcellulose (10 wt%), and a solvent (deionized water). This blend was evenly spread over Cu foil and wholly dried under vacuum at 90 °C for 10 h. Furthermore, the mean mass loading of the active substances varied between 0.6 and 1.2 mg cm^−2^. A self-made Na metal foil was utilized as the opposing electrode, with Whatman glass fiber (GF/D) acting as the isolator, and 1 M NaPF_6_ in ethylene glycol dimethyl ether (DME) as the electrolytic solution. The battery cells were constructed inside a glove compartment filled with highly pure argon gas (O_2_ and H_2_O levels < 0.01 ppm). The galvanostatic charge/discharge patterns were assessed using a LAND-CT3002A battery evaluation system (Wuhan LAND Electronic Co., Ltd., Wuhan, China) within the range of 0.01–3 V (compared to Na^+^/Na). The cyclic voltammetry (CV) experiments were conducted using an electrochemical workstation (CHI650E, Instrument Co., Ltd., Shanghai, China) at varying scan rates. Electrochemical impedance spectroscopy (EIS) was carried out with a frequency span of 0.01–100 kHz on the CHI650E electrochemical workstation.

## 3. Results and Discussion

**Figure 1:**
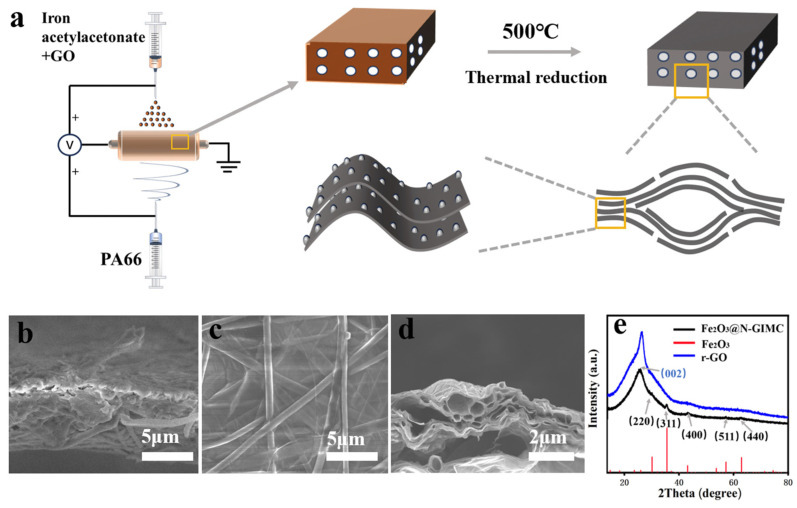
(**a**) Preparation of composite materials. The scanning electron microscope (SEM) of Fe_2_O_3_@G (**b**) and Fe_2_O_3_@N-GIMC (**c**,**d**); (**e**) the X-ray diffraction (XRD) of Fe_2_O_3_@N-GIMC and r-GO.

Many of our previous works have shown that electrospray is an effective technology to assemble GO film using GO dispersion [42,43,44,45]. The r-GO film can be obtained by high temperature reduction. Here, to prepare the r-GO/Fe_2_O_3_ composite material, a mixed solution of GO and iron acetylacetonate was first prepared as a precursor solution. And, then, the mixed solution was loaded into a syringe to prepare GO film containing iron acetylacetonate by using electrospray technique, as described in Figure 1a. At the same time, nylon nanofibers were prepared by electrospinning technology on the other side of the collector electrode. The integration of electrospinning and electrospray technology was employed to effectively fabricate the composite film of GO flakes with iron acetylacetonate and nylon nanofibers, as shown in Figure 1b. Following the preparation, the combined film underwent further annealing at a high temperature. In this process, GO was reduced, iron acetylacetonate was decomposed to produce iron oxide, nylon fiber was decomposed entirely at high temperature, and finally successfully prepared Fe_2_O_3_@N-GIMC, as shown in Figure 1c,d. It is obvious that with the decomposition of the nylon nanofibers, well-shaped and interconnected micro-channels are formed inside the film. The arrangement of the composite material prepared in this way was analyzed using X-ray diffraction (XRD) (Figure 1e), it shows XRD signals that can all be indexed to Fe_2_O_3_ (JCPDS No. 39-1346), indicating that the composition of the composite sample does contain Fe_2_O_3_. It exhibits broad reflection peaks without any distinct peaks, indicating the presence of iron oxide nanoparticles in an amorphous state [46,47]. The diffraction peak at (0 0 2) of the hybrid material originates from reduced graphene oxide (r-GO); this is consistent with the XRD peaks of r-GO.

**Figure 2:**
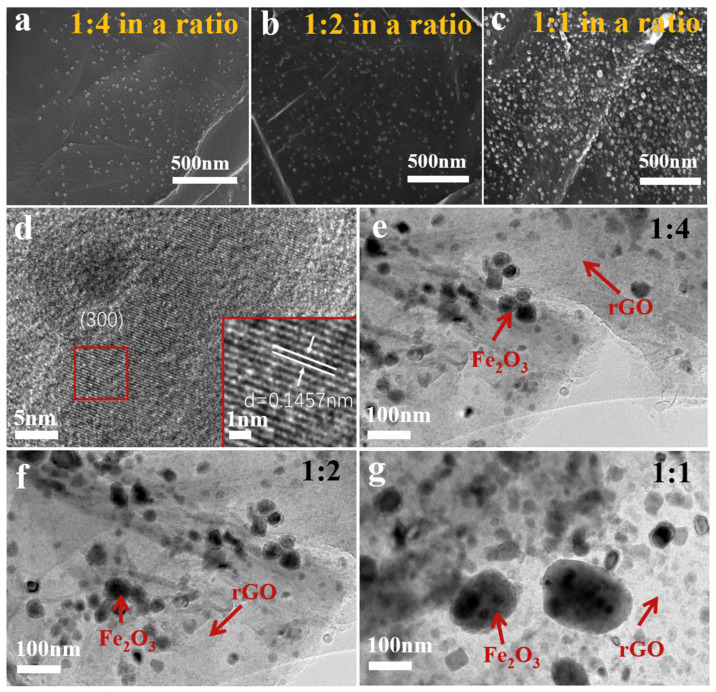
(**a**–**c**) SEM image of Fe_2_O_3_@N-GIMC; (**d**) High Resolution Transmission Electron Microscope (HRTEM) image of Fe_2_O_3_@N-GIMC; (**e**–**g**) Transmission Electron Microscope (TEM) images of Fe_2_O_3_@N-GIMC.

As shown in Figure S1 and Figure 2a–c, the particles of Fe_2_O_3_ were obviously formed on the r-GO surface inside the sample. Elements C, Fe, O, and N are evenly spread throughout the area. Among them, the N element comes from the high-temperature decomposition of nylon nanofibers. With the increase of the proportion of iron acetylacetonate content in the precursor solution, more and more Fe_2_O_3_ particles are formed, and the corresponding Fe element is also increasing. High Resolution Transmission Electron Microscope (HRTEM) and Transmission Electron Microscope (TEM) of Fe_2_O_3_@N-GIMC were measured for further study of the microstructure of Fe_2_O_3_@N-GIMC. The HRTEM image (Figure 2d) shows that lattice streaks of the composite with (300) spacing can be observed, which does not correspond to the lattice structure of Fe_2_O_3_. In addition, the XRD pattern of Fe_2_O_3_@N-GIMC (Figure 1e) shows that the characteristic diffraction peak of Fe_2_O_3_ is not obvious, and the spectral peak is widened. In general, amorphous structures are composed of extremely small (<2 nm) grain composition, the XRD image will be diffused and broadened. Therefore, it can be inferred that the Fe_2_O_3_@N-GIMC particle has a low crystallinity and an amorphous structure. Based on the depiction in Figure 2e–g, a clear distinction between the Fe_2_O_3_ and graphene phases can be discerned. It can be obviously seen that the size ranges of Fe_2_O_3_ phases in Fe_2_O_3_@N-GIMC_1:4, Fe_2_O_3_@N-GIMC_1:2, and Fe_2_O_3_@N-GIMC_1:1 are 20 nm~50 nm, 20 nm~60 nm, and 20 nm~210 nm, respectively. It can be seen with the increase of the amount of iron acetylacetonate doping, the size of Fe_2_O_3_ phases in Fe_2_O_3_@N-GIMC_1:2 is almost the same as Fe_2_O_3_@N-GIMC_1:4, and the Fe_2_O_3_ phases of both is uniformly dispersed. Still, the Fe_2_O_3_ phases density of 1:2 is greater on the graphene sheet per unit area. As the doping amount of iron acetylacetonate continues to increase, there is a serious aggregation of Fe_2_O_3_ phases in Fe_2_O_3_@N-GIMC_1:1, which also causes the size of Fe_2_O_3_ phases to grow to 210 nm.

**Figure 3:**
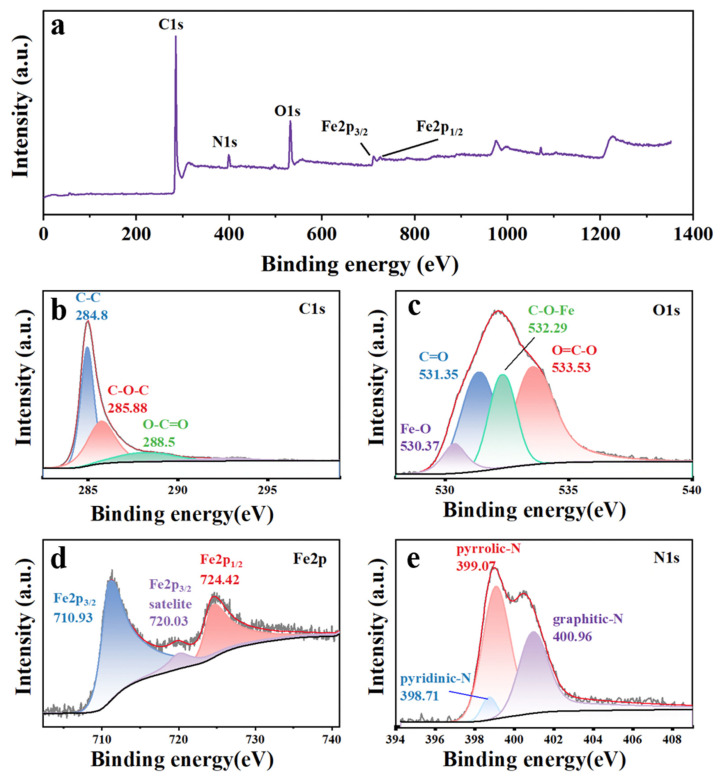
X-ray Photoelectron Spectroscopy (XPS) spectra of Fe_2_O_3_@N-GIMC. (**a**) XPS survey spectra; (**b**) C1s; (**c**) O1s; (**d**) Fe2p; (**e**) N1s.

X-ray Photoelectron Spectroscopy (XPS) measurements were meticulously employed to gain profound insights into the surface chemistry of Fe_2_O_3_@N-GIMC, effectively unveiling a noteworthy nitrogen weight content of up to 5.65%, which represents a moderate doping concentration (Figure 3a). As illustrated in Figure 3b, the C1s spectrum of GF is mainly composed of three kinds of bonds: C-C (284.8 eV), C-O-C (285.88 eV), and O-C=O (288.5 eV). This shows that the C element has been reduced [42]. To further prove the interaction between the keys, the O1s map was analyzed, as shown in Figure 3c. It is mainly composed of five types of keys: Fe-O (530.37 eV), C=O (531.35 eV), C-O-Fe (532.29 eV), C-O/O=C-O (533.53 eV), Fe-O (530.37 eV), which more indirectly proved the presence of iron trioxide in the composite material. The existence of C-O-Fe (532.29 eV) chemical bonds is compelling evidence for the robust interconnection between iron trioxide and graphene [37]. The Fe2p spectrum and the results of their fitting are displayed in Figure 3d. The spectrum revealed the presence of doublet Fe2p_3/2_ and Fe2p_1/2_ with binding energies of 711.25 eV and 724.7 eV, correspondingly. The Fe2p_3/2_ peak was also accompanied by a satellite peak positioned at 720.03 eV, which was indicative of α-Fe_2_O_3_ and aligned closely with the values reported in existing literature [48]. It indicates the presence of ferric oxide in r-GO, indirectly proving that the reaction was successful [37]. Most notably, XPS spectra demonstrate the robust interfacial connection between Fe_2_O_3_ and r-GO in the r-GO/Fe_2_O_3_ composite material. Figure 3e shows that the N1s spectrum consists of three peaks at 398.71 eV, 399.07 eV, and 400.96 eV, corresponding to pyridine-N, pyrrole-N, and graphite-N, respectively [49]. This shows that N doping of graphene sheets has been successfully achieved.

**Figure 4:**
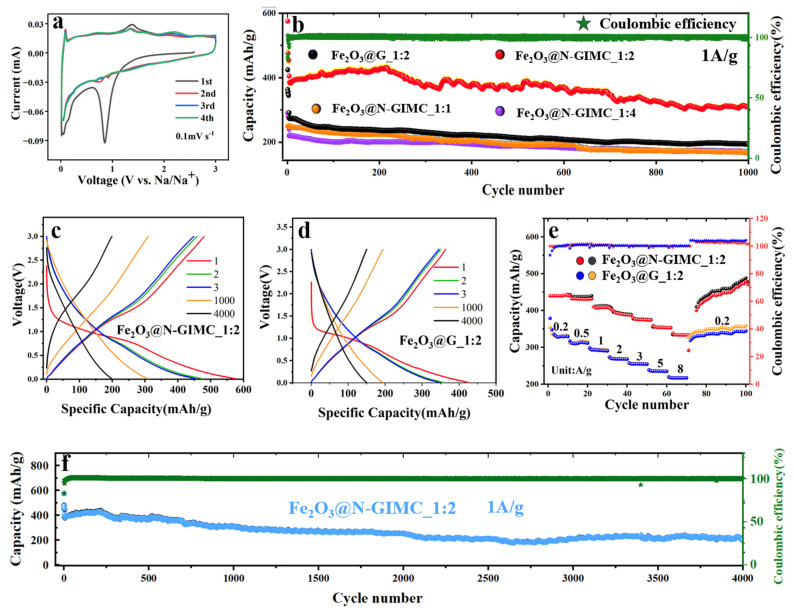
(**a**) CV plots at a scan rate of 0.1 mV s^−1^ for Fe_2_O_3_@N-GIMC_1:2; (**b**) cycle stability and electrochemical efficiency for Fe_2_O_3_@N-GIMC_1:4, Fe_2_O_3_@N-GIMC_1:2, Fe_2_O_3_@N-GIMC_1:1, and Fe_2_O_3_@G_1:2 cycled at a current of 1 A g^−1^. Initial five discharge–charge curves for Fe_2_O_3_@N-GIMC_1:2; (**c**) and Fe_2_O_3_@G_1:2; (**d**) at 1 A g^−1^; (**e**) rate capability of Fe_2_O_3_@N-GIMC_1:2 and Fe_2_O_3_@G_1:2; (**f**) cycling performance for Fe_2_O_3_@N-GIMC_1:2 cycled at a current of 1 A g^−1^.

To investigate the sodium-ion insertion/extraction mechanisms in the Fe_2_O_3_@N-GIMC_1:2 electrode, cyclic voltammetry (CV) was employed, as depicted in Figure 4a. In the primary cycle, two distinct reduction peaks at around ~0.01 V and 0.85 V are associated with Na penetration into Fe_2_O_3_@N-GIMC_1:2 and the creation of the SEI layers, respectively. Following the vanishing of the peak at 0.85 V, indicative of the establishment of enduring solid electrolyte interphase (SEI) layers, enhanced cyclic durability is evidenced [50]. In the anodic CV curves of Fe_2_O_3_@N-GIMC_1:2, two discernible peaks at approximately 0.08 V and 1.37 V are observed, corresponding to the extraction of sodium from Fe_2_O_3_@N-GIMC_1:2 and the corrosion of certain reversible solid electrolyte interface components. In the second CV curve, the cathode peaks at 0.76 and 0.92 V are accompanied by anode peaks at 0.72 and 1.36 V, which correspond to the reversible redox reaction of Fe^3+^↔Fe^2+^, Fe^2+^↔Fe^0^, and Fe dissolution [51,52]. The cyclic voltammetry plots exhibit almost perfect overlap from the second cycle onwards, underscoring the exceptional electrochemical reversibility of the Fe_2_O_3_@N-GIMC_1:2 electrode.

The Fe_2_O_3_@N-GIMC_1:2 electrode demonstrates remarkable cycling stability and high Coulombic efficiency, as illustrated in Figure 4b. Following 1000 cycles at a current density of 1 A g^−1^, the discharge capacity retention reached 67%, surpassing the values of 65% and 48% observed for Fe_2_O_3_@N-GIMC_1:1 and Fe_2_O_3_@N-GIMC_1:4, respectively. This is because, since a small amount of Fe_2_O_3_ doping can only slightly improve the electrochemical performance, while excessive doping causes Fe_2_O_3_ agglomeration (this is confirmed in Figure 2g). So, the excellent electrochemical performance of Fe_2_O_3_@N-GIMC_1:2 should be attributed to the right amount of Fe_2_O_3_ doping. Moreover, after undergoing 1000 cycles at a current density of 1 A g^−1^, Fe_2_O_3_@N-GIMC_1:2 demonstrates an impressive discharge capacity retention of 67%, outperforming Fe_2_O_3_@G_1:2 which only maintains 53% of its initial discharge capacity. Similarly, after enduring 4500 cycles at the same current density, Fe_2_O_3_@N-GIMC_1:2 exhibits a remarkable discharge capacity retention of 51%, while Fe_2_O_3_@G_1:2 only manages to retain 45%. These results highlight the excellent long-term cycle stability of Fe_2_O_3_@N-GIMC_1:2. The Fe_2_O_3_@N-GIMC_1:2 demonstrates remarkable discharge capacity (573.5 mAh g^−1^ at 1 A g^−1^) as a sodium-ion batteries anode (Figure 4c), and that of Fe_2_O_3_@G_1:2 only 424.6 mAh g^−1^ (Figure 4d). This phenomenon can be attributed to the enhanced provision of ways of transmission and sites of absorption by the internal channels. At the same time, the interconnected architecture effectively reduces the pulverization and aggregation of electrode-related materials. Furthermore, N doping is also beneficial to improve its electrochemical performance. In Figure 4e, the rate performance of the two samples at various current densities is presented. The discharge-specific capacities of Fe_2_O_3_@N-GIMC_1:2 at current densities of 0.2, 0.5, 1, 2, 3, 5, and 8 A g^−1^ are measured at 440.6 mAh g^−1^, 430.3 mAh g^−1^, 408.0 mAh g^−1^, 389.7 mAh g^−1^, 374.1 mAh g^−1^, 354.3 mAh g^−1^, and 333.6 mAh g^−1^, respectively. Upon returning the current density to 0.2 A g^−1^, the discharge specific capacities of Fe_2_O_3_@N-GIMC_1:2 could revert to the initial level of 454.2 mAh g^−1^, demonstrating excellent tolerance to high currents. Overall, in comparison to Fe_2_O_3_@G_1:2, the rate performance of Fe_2_O_3_@N-GIMC_1:2 exhibits superiority over Fe_2_O_3_@G_1:2. Moreover, the Fe_2_O_3_@N-GIMC_1:2 electrode still maintains a high specific discharge capacity after a long cycle of 4000 cycles (Figure 4f). In conclusion, despite an equivalent Fe doping level in Fe_2_O_3_@N-GIMC_1:2 and Fe_2_O_3_@G_1:2, the Fe_2_O_3_@N-GIMC_1:2 electrode demonstrates superior initial coulombic efficiency, exceptional reversibility, improved rate performance and excellent cycling stability in sodium-ion batteries. On one side, the three-dimensional interconnected structure can effectively prevent fragmentation and clustering of electrode materials because of their exceptional structural integrity. It leads to fast ion transport and efficient use of active materials, thereby enhancing the speed performance and longevity. On the flip side, the multiple hollow channels can offer additional routes for ion transmission and sites for absorption of sodium ions, as well as reduce transmission distance, resulting in a higher capacity.

**Figure 5:**
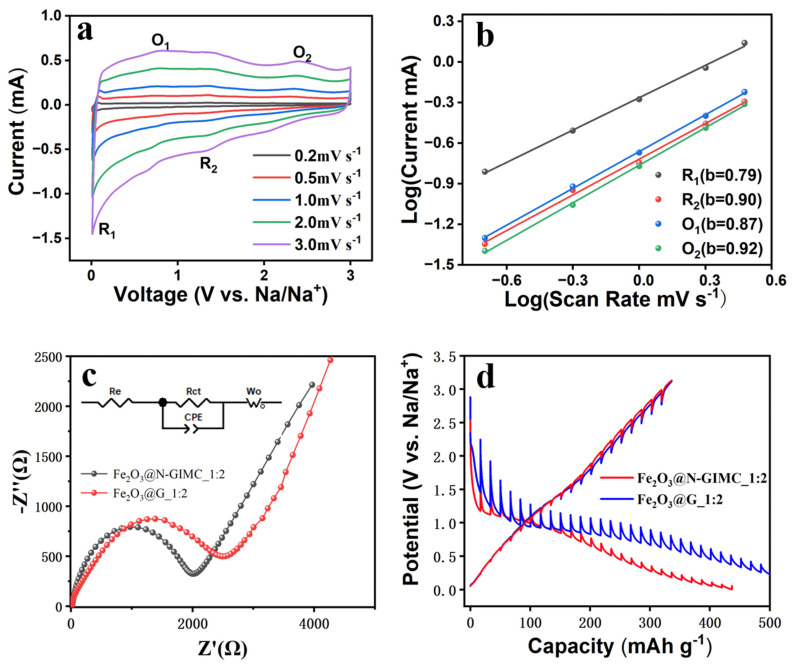
(**a**) CV curves of Fe_2_O_3_@N-GIMC_1:2 at various scan rates from 0.2 to 3 mV s^−1^; (**b**) log (i) versus log (v) plots; (**c**) electrochemical impedance spectroscopy (EIS) of the Fe_2_O_3_@N-GIMC_1:2 electrode and Fe_2_O_3_@G_1:2 electrode; (**d**) galvanostatic intermittent titration technique (GITT) potential profiles of Fe_2_O_3_@N-GIMC_1:2 and Fe_2_O_3_@G_1:2 electrode.

To comprehend the reaction speed as well as the reversible capabilities and extended cycle longevity of the Fe_2_O_3_@N-GIMC_1:2, cyclic voltammetry plots at different scanning speeds were gathered. (Figure 5a). The scan rates are increased from 0.2 to 3 mV s^−1^, similar outlines and slight peak shifts at different scan rates are illustrated by the excellent reversibility and small polarizations of Fe_2_O_3_@N-GIMC_1:2 in Figure 5a. Furthermore, based on the cyclic voltammetry plots, the correlation between the peak current (i) and the scan rate (v) complies with Equation (1):

i = av^b^ (1)

where a is a constant, and b can be ascertained by the gradients of log (i) versus log (v). If b equals 0.5, it signifies that the electrochemical process is governed by a diffusion-controlled mechanism; if b equals 1, the electrochemical process is dictated by surface-responsive capacity behavior. The b-values measured for the Fe_2_O_3_@N-GIMC_1:2 electrode (Figure 5b) is located within a range between 0.79 and 0.92, Their b values were all near 1, suggesting a surface capacitance-driven behavior, resulting in a swift Na^+^ intercalation/deintercalation process and excellent rate capability.

The internal energy storage mechanism of Fe_2_O_3_@N-GIMC_1:2 as the anode material for sodium-ion batteries (SIBs) was further elucidated through electrochemical impedance spectroscopy (EIS) analysis, as shown in Figure 5c. The Nyquist plot is characterized by the presence of a semicircular arc intersecting the graph, along with an accompanying linear segment. The semicircular region observed in the high-frequency domain of the Nyquist plot signifies the electron transfer-limited mechanism, while the linear portion evident in the low-frequency range denotes the diffusion-limited process. In the faster electron transport process, the AC impedance spectrum contains only a straight line portion, while the slower charge transport process has a larger semicircular region. The diameter of the semicircle corresponds to the charge transfer resistance (Rct), while the intercept (Zre) of the semicircle on the real axis represents the electrolyte resistance (Re). The fitting results are shown in Table 2 according to the equivalent circuit diagram. The Rct of the Fe_2_O_3_@N-GIMC_1:2 electrode is lower than Fe_2_O_3_@G_1:2 electrode. This suggests a more rapid charge transfer between the Fe_2_O_3_@N-GIMC_1:2 electrode surface. This shows that in the electrochemical process of the negative electrode of SIB, the channel of Fe_2_O_3_@N-GIMC_1:2 can shorten the ion diffusion distance and accelerate the ion diffusion, and the doping of N atom is conducive to improving the conductivity and electrochemical activity of Fe_2_O_3_@N-GIMC_1:2. Moreover, to reveal the detailed reaction kinetics for the Na^+^ intercalation/deintercalation processes, the galvanostatic intermittent titration technique (GITT) was performed (Figure 5d). The diffusion coefficient (D) of Fe_2_O_3_@N-GIMC_1:2 can be calculated from the GITT potential profiles using Fick’s second law with the following Equation (2):

(2) $$D=\frac{4}{\Pi \tau }(\frac{m_{B}}{M_{b}}\frac{V_{M}}{S}{)}^{2}(\frac{\Delta E_{s}}{\Delta E_{t}}{)}^{2}$$

where *τ* represents the duration of the current pulse; *m_B_* represents the mass loading of the electrode material; *S* represents the geometric area of the electrode; ∆*E_s_* is the quasi-thermodynamic equilibrium potential difference between before and after the current pulse; ∆*E_t_* is the potential difference during the current pulse; *V_M_* is the molar volume of the materials; and *M_b_* is the molar mass of Fe_2_O_3_@N-GIMC_1:2 materials and the results are plotted as a function of electrochemical potential. As shown in Figure S2, Fe_2_O_3_@N-GIMC_1:2 delivers a high D-value at almost all potentials, resulting from reinforced effects of the abundant heterointerfaces, porous, and interconnected C skeleton.

## 4. Conclusions

In summary, the synthesis of Fe_2_O_3_ anchored onto nitrogen-doped graphene with internal micro-channels (Fe_2_O_3_@N-GIMC) was effectively achieved. The synthesized Fe_2_O_3_@N-GIMC_1:2 demonstrates remarkable discharge capacity (573.5 mAh g^−1^ at 1 A g^−1^), notable rate capability (333.6 mAh g^−1^ at 8 A g^−1^), and consistent long-term cycle stability (308.9 mAh g^−1^ after 1000 cycles at 1 A g^−1^, 200.8 mAh g^−1^ after 4000 cycles at 1 A g^−1^) as a sodium-ion battery anode. Following 1000 cycles under a current density of 1 A g^−1^, the Fe_2_O_3_@N-GIMC_1:2 composite retained 67% of its discharge capacity, while the Fe_2_O_3_@G_1:2 composite exhibited a lower capacity retention of 53%. This phenomenon can be ascribed to the enhanced availability of transmission pathways and absorption sites facilitated by the internal channels, alongside the interconnected architecture’s efficacy in mitigating electrode material pulverization and aggregation. In addition, N doping is also beneficial to improve its electrochemical performance. This offers a novel approach for producing graphene-based advanced composites and establishes a basis for broadening their applications.

## Figures and Tables

**Table 1:** Technical specifications of GO.

Model Number	Single Layer Rate	Transverse Size Distribution	Particle Size (D50)	Viscosity (cp, 10 mg/g)
GO2-DMF	>99%	20–30 μm	1.9 ± 0.2 μm	4800 ± 8000

**Table 2:** EIS fitting results of Fe_2_O_3_@N-GIMC_1:2 electrode and Fe_2_O_3_@G_1:2 electrode before cycling.

	Re/Ω	Rct/Ω	CPE1-T/Ω	CPE1-P/Ω	W1-R/Ω	W1-T/Ω	W1-P/Ω
Fe_2_O_3_@N-GIMC_1:2	15.4	1842	0.000000087	0.87	1182	18.11	0.52
Fe_2_O_3_@G_1:2	14.1	2087	0.000000045	0.64	1803	17.20	0.49

## Data Availability

Data will be made available on request.

## Supplementary Materials

The following supporting information can be downloaded at: https://www.mdpi.com/article/10.3390/nano14110937/s1, Figure S1: The Energy Dispersive Spectrometer (EDS) of Fe_2_O_3_@N-GIMC_1:4, Fe_2_O_3_@N-GIMC_1:2 and Fe_2_O_3_@N-GIMC_1:1; Figure S2: Diffusion coefficients of Fe_2_O_3_@N-GIMC_1:2.
