# Supplementary material for: Amorphous Fe2O3 Anchored on N-Doped Graphene with Internal Micro-Channels as an Active and Durable Anode for Sodium-Ion Batteries
Source: Nanomaterials (Basel). 2024 May 27;14(11):937. doi: 10.3390/nano14110937 (PMC11173887; doi:10.3390/nano14110937)
Supplement: Supplementary file 1 [file nanomaterials-14-00937-s001.zip › nanomaterials-3010077-supplementary.pdf]

Supporting information

Amorphous Fe_2O_3 anchored on N-doped graphene with internal micro-channels as an active and durable anode for sodium-ion batteries

Lin Li[1†], Hui Li[2†], Linxin Liu[1], Xunchang Yan[1], Yunze Long[1,2,*], Wenpeng Han[1,2,*]

[1] Collaborative Innovation Center for Nanomaterials & Devices, College of Physics, Qingdao University, Qingdao 266071, China

[2] State Key Laboratory of Bio-Fibers and Eco-Textiles, Qingdao University, Qingdao 266071, China

* Corresponding Authors.

E-mail addresses: yunze.long@qdu.edu.cn (Y.L.); wphan@qdu.edu.cn (W.H.)
† These authors contributed equally to this work.

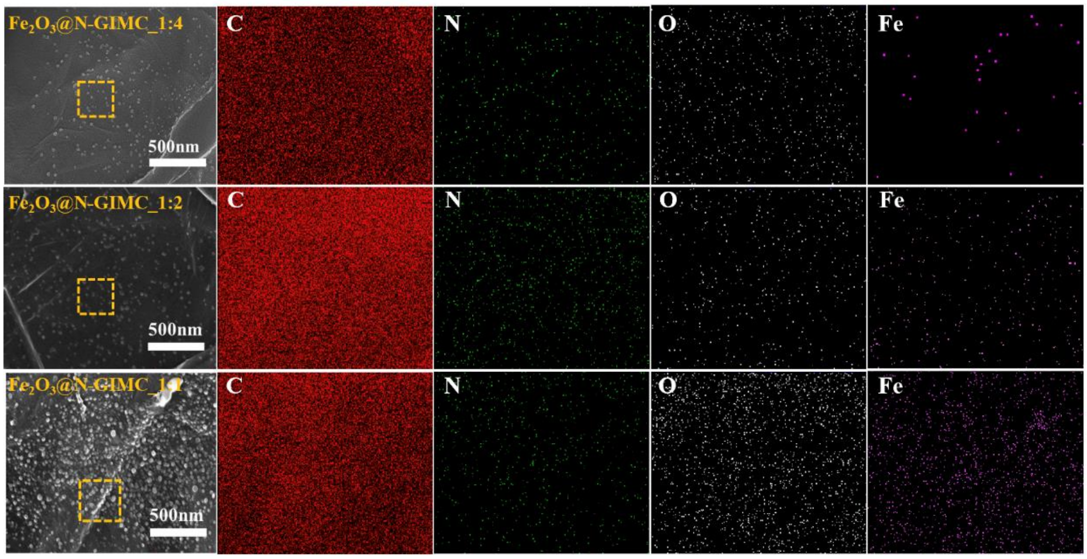

Figure S1. The Energy Dispersive Spectrometer (EDS) of Fe_2O_3@N-GIMC_1:4, Fe_2O_3@N-GIMC_1:2 and Fe_2O_3@N-GIMC_1:1.

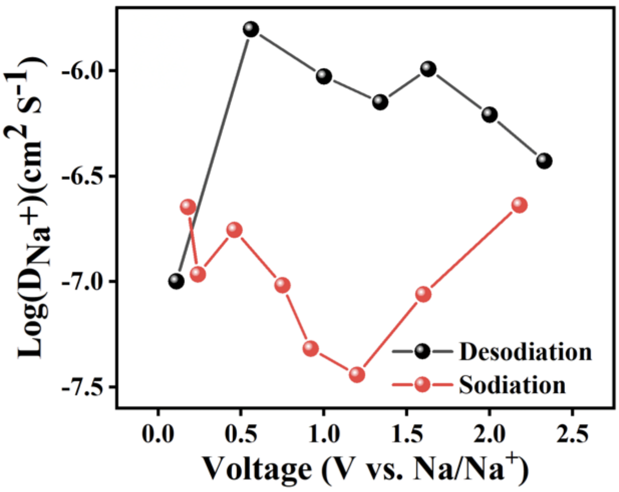

Figure S2. Diffusion coefficients of Fe_2O_3@N-GIMC_1:2.
